# HP1 Recruits Activity-Dependent Neuroprotective Protein to H3K9me3 Marked Pericentromeric Heterochromatin for Silencing of Major Satellite Repeats

**DOI:** 10.1371/journal.pone.0015894

**Published:** 2011-01-18

**Authors:** Kerstin Mosch, Henriette Franz, Szabolcs Soeroes, Prim B. Singh, Wolfgang Fischle

**Affiliations:** 1 Laboratory of Chromatin Biochemistry, Max Planck Institute for Biophysical Chemistry, Göttingen, Germany; 2 Division of Immunoepigenetics, Research Center Borstel, Borstel, Germany; National Institute on Aging (NIA), National Institutes of Health (NIH), United States of America

## Abstract

H3 lysine 9 trimethylation (H3K9me3) is a histone posttranslational modification (PTM) that has emerged as hallmark of pericentromeric heterochromatin. This constitutive chromatin domain is composed of repetitive DNA elements, whose transcription is differentially regulated. Mammalian cells contain three HP1 proteins, HP1α, HP1β and HP1γ These have been shown to bind to H3K9me3 and are thought to mediate the effects of this histone PTM. However, the mechanisms of HP1 chromatin regulation and the exact functional role at pericentromeric heterochromatin are still unclear. Here, we identify activity-dependent neuroprotective protein (ADNP) as an H3K9me3 associated factor. We show that ADNP does not bind H3K9me3 directly, but that interaction is mediated by all three HP1 isoforms *in vitro*. However, in cells ADNP localization to areas of pericentromeric heterochromatin is only dependent on HP1α and HP1β. Besides a PGVLL sequence patch we uncovered an ARKS motif within the ADNP homeodomain involved in HP1 dependent H3K9me3 association and localization to pericentromeric heterochromatin. While knockdown of ADNP had no effect on HP1 distribution and heterochromatic histone and DNA modifications, we found ADNP silencing major satellite repeats. Our results identify a novel factor in the translation of H3K9me3 at pericentromeric heterochromatin that regulates transcription.

## Introduction

The physiological template of genetic information in all eukaryotic cells is chromatin. As biological relay station and signaling platform chromatin integrates a variety of endogenous and exogenous cellular inputs. The various signals are thought to direct distinct local and global functional states of chromatin, therefore controlling the capacity of a cell's genome to store, release, and inherit biological information. In the repeating unit of chromatin, the nucleosome, DNA is wrapped around an octamer of core histone proteins (two copies each of H2A, H2B, H3, and H4).

On a cytological level, functionally different types of chromatin have been described: euchromatin appears less condensed, is thought to have a more “open” conformation, contains the majority of genes, replicates early and throughout S-phase, and is mostly transcriptionally active. Heterochromatin, in contrast, appears condensed with relative even spacing of nucleosomes, is thought to have a more “closed” conformation, comprises few genes, is replicating late in S-phase, and is largely transcriptionally silent (for review see [1]. Distinct regions of facultative heterochromatin are characteristic for differentiated cells, while constitutive heterochromatin is found in all cells at specific chromosomal territories, namely telomeres, centromeres and pericentromeric regions. The latter are essential for chromosome replication containing repetitive satellite sequences. In *S. pombe* transcription from such regions is involved in the establishment and maintenance of the heterochromatin state implicating an RNAi dependent mechanism [2], [3], [4]. In higher eukaryotes centromeric and pericentromeric transcription varies during cellular development and differentiation [5]. However, the exact mechanism(s) by which the activity of these elements is regulated has not been fully elucidated.

Besides regulated incorporation of histone variants, the architecture of chromatin on the nucleosomal level is essentially the same for all of the genome. Divergence is achieved via regional restricted methylation of DNA as well as numerous post-translational modifications of the histone proteins (PTMs). These include acetylation and mono-(me1), di-(me2) and tri-(me3) methylation of numerous lysine residues in all four core histones. The high complexity of histone PTMs gives an enormous potential for differential functional responses [6]. Transcriptionally active areas of the genome are largely associated with global hyperacetylation of histone lysine residues as well as site specific methylation of H3 lysine 4 (H3K4me) and H3 lysine 36 (H3K36me) [7], [8]. On the other hand, pericentromeric heterochromatin in higher eukaryotes is enriched in H3 lysine 9 trimethylation (H3K9me3), H4 lysine 20 trimethylation (H4K20me3) as well as H3 lysine 27 monomethylation (H3K27me1). Linkage to H3 arginine 2 dimethylation (H3R2me2) and H4 arginine 3 dimethylation (H4R3me2) has also been described [9], [10]. Especially, H3K9me2/3 has been recognized as a hallmark of heterochromatin [8]. In *S. pombe* it could be shown that this PTM is central to establishment and maintenance of heterochromatin at the centromeric but also mating type regions [3], [4], [11]. Nevertheless, the molecular and functional relation of the histone PTM status and particular chromatin structures at heterochromatin has not been fully described.

Di- and trimethylation of histone H3 lysine 9 are mediated by the Suv39h1/h2 isoenzymes, CLLD8/KMT1F as well as the ESET/SETDB1 histone methyltransferase [12], [13], [14]. SETDB1 is mainly found in euchromatic regions, where it participates in gene silencing [15]. In contrast, CLLD8/KMT1F and Suv39-like enzymes localize to pericentromeric heterochromatin [14]. Suv39h1^−^/^−^,Suv39h2^−^/^−^ double knockout MEF cells fail to show H3K9me3 at these loci indicating that Suv39h1/h2 are the main HMTases establishing H3K9 trimethylation at pericentromeric regions [9]. The level of transcripts from the major satellite repeats is slightly upregulated in Suv39h1^−^/^−^,Suv39h2^−^/^−^ double knockout MEF cells indicating a potential role of H3K9me3 in transcriptional silencing at pericentromeric heterochromatin. It has been suggested that reduction in DNA methylation levels is causally involved in these events [16].

Another essential component of Suv39 control mechanisms at pericentromeric heterochromatin is heterochromatin protein 1 (HP1). Mammalian cells contain three isoforms HP1α, HP1β and HP1γ, which all localize to pericentromeric heterochromatin to different degrees [17]. HP1 proteins consist of an N-terminal chromo domain (CD), a flexible hinge region and a C-terminal chromoshadow domain (CSD). The CD of HP1 is a binding module for H3K9me3 [18], [19], [20]. Interestingly, the amino acid context of H3K9 (“ARKS”) is found in identical or similar form at multiple other sites in histones and other proteins. The corresponding lysines have been found to be methylated in several of these instances: H3K27: ARKmeS binds Polycomb and to a lesser degree HP1 [21], H1K26: ARKmeS binds HP1 [22], G9aK165: ARKmeT binds HP1 [23].

The HP1 CSD mediates homodimerization with the same HP1 isoform as well as heterodimerization between different HP1 isoforms [24], [25], [26], [27]. A phage display screen identified a pentapeptide motif PxVxL (x =  any amino acid) that interacts specifically with the HP1 CSD dimer [28]. This peptide motif is present in a number of HP1 interaction partners, for example in KAP-1 [24], Suv37 [29], CAF-1 p150 [24], the TAFII130 component of TFIID [30], and AF10 [31]. Also, Suv39h1 itself interacts with HP1 via the CSD [32], [33]. Binding of HP1 to a variety of interaction partners such as histone methyltransferases and transcriptional repressors suggests HP1 could serve as a platform for the recruitment of downstream factors to H3K9me3 [16], [24], [34]. It is thought that such factors then indirectly mediate the translation of H3K9me3.

Activity-dependent neuroprotective protein (ADNP) was originally cloned from mouse carcinoma cells, differentiated into neuroglial cells [35]. The predicted structure of the 124 kDa protein contains nine zinc fingers, a proline-rich region, a nuclear bipartite localization signal, cellular export and import signals and a homeobox domain profile. Extra- and intracellular ADNP functions have been described [36]. The protein is secreted from glial cells and constitutes part of the vasoactive intestinal peptide (VIP)-stimulated neuron protective pathway [37]. Neuroprotection of ADNP is conferred by NAP, an 8-amino acid fragment of ADNP that might target tubulin in astrocytes after internalization [35], [38], [39], [40], [41]. In the cell nucleus ADNP has been identified as one of the proteins that interact with the interferon-alpha promoter [42]. Down-regulation of ADNP up-regulates the tumor suppressor p53 and reduces cell viability [43]. Analysis in ADNP knockout mice reveals failure of cranial neural tube closure and death on E8.5–9.5 [44]. More than 200 factors were found transcriptionally up or down regulated in absence of ADNP [45]. Further, co-immunoprecipitation experiments identify components of the SWI/SNF (mating type switching/sucrose nonfermenting) complex, as interaction partners of ADNP [46]. However, the exact chromatin roles of ADNP have not been elucidated.

## Results

### Identification of ADNP as H3K9me3 associated protein

**Figure 1:**
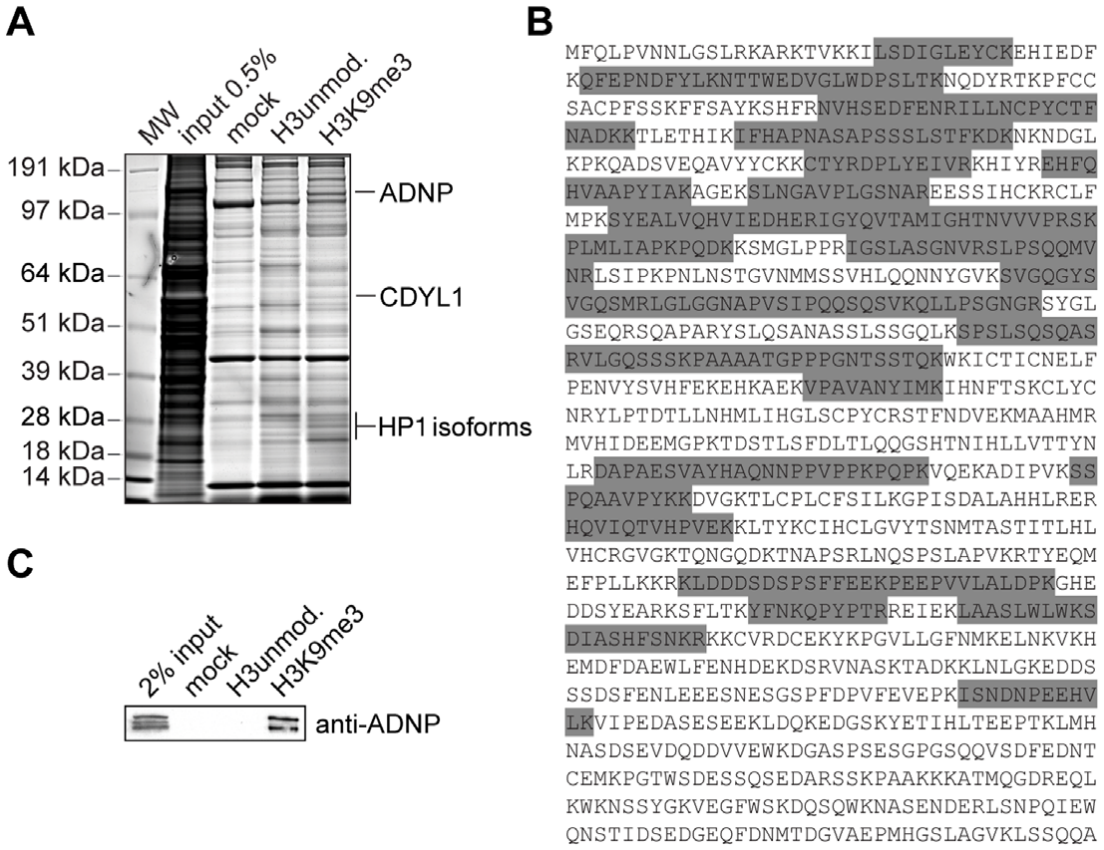
Identification of ADNP as an H3K9me3 associated factor. (**A**) The indicated histone H3 peptides were used in pulldown experiments of HeLa S3 cell nuclear extract. Beads without coupled peptides were used as control (mock). Specifically recovered proteins were run on SDS-PAGE and stained with Coomassie Blue. Proteins were identified by MS/MS analysis. The positions of ADNP, HP1 and CDYL1 proteins are indicated on the right. Molecular weight (MW) markers are indicated on the left. (**B**) Amino acid sequence of ADNP. The highlighted (grey) peptides were identified by MS/MS analysis. (**C**) Western blot analysis of a histone H3 peptide pulldown experiment of HeLa S3 cell nuclear extract as in (A) using an anti-ADNP antibody.

To gain insights into heterochromatin formation and maintenance we used a histone peptide pulldown approach. To identify proteins interacting with the H3K9me3 histone PTM H3 (aa 1-21) peptides unmodified or trimethylated at lysine 9 were immobilized on magnetic beads and incubated with HeLa S3 nuclear extract. Proteins bound to the different affinity matrices were separated by SDS-PAGE. Whole lanes were cut into pieces, digested with trypsin and subjected to MS/MS analysis (Figure 1A). Full sets of factors from the different pulldowns were subtracted yielding proteins specifically interacting with the H3 tail depending on the K9me3 modification. Among the factors identified in such experiments were several known H3K9me3 interaction partners like the three isoforms of HP1, HP1αHP1β and HP1γ as well as CDYL1 [47]. One factor that had so far not been described in the context of heterochromatin or H3K9me3 was the activity dependent neuroprotector protein, ADNP. As Figure 1B shows a total of 36 (partially overlapping) peptides of this H3K9me3 interacting protein could be identified (overall sequence coverage of 43%).

To independently confirm the results of the MS analysis, we repeated the H3 peptide pulldowns from HeLa S3 nuclear extracts. Using a monoclonal antibody in western blotting experiments we could confirm specific enrichment of ADNP exclusively on the H3K9me3 matrix compared to the unmodified peptide and the mock control (Figure 1C; see Figure S2 for antibody characterization).

### ADNP associates with H3K9me3 in chromatin context

**Figure 2:**
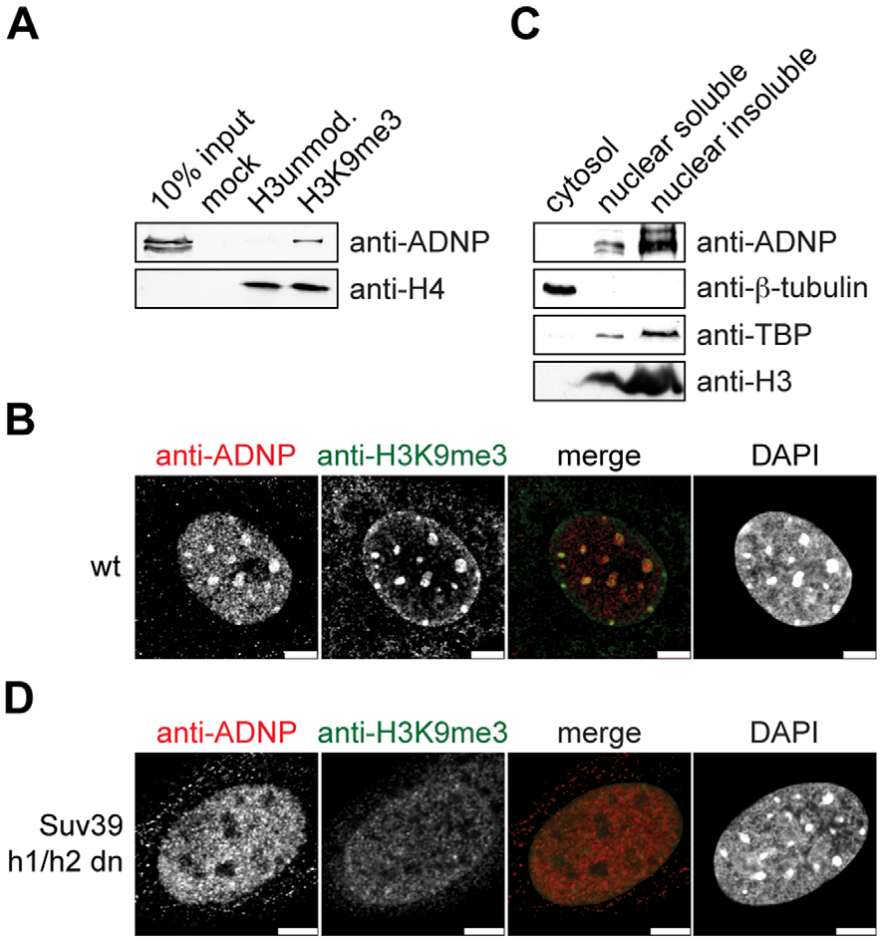
ADNP associates with H3K9me3 chromatin. (**A**) Recombinant 12-mer oligonucleosomal chromatin arrays reconstituted with the indicated H3 species were immobilized on magnetic beads and incubated with HeLa S3 nuclear extract. Beads without coupled oligonucleosomes were used as control (mock). Western blots of the pulldown reactions using the indicated antibodies are shown. (**B**) Immunofluorescence analysis of wild type MEF cells using the indicated antibodies. DNA was visualized using DAPI. Bars, 5 µm. (**C**) NIH3T3 cells were subjected to subcellular fractionation. The cytoplasmatic fraction was extracted with Triton X-100. Nuclei were lysed under salt-free conditions. Soluble and insoluble fractions were separated by centrifugation and analyzed by western blotting using the indicated antibodies. (**D**) Immunofluorescence analysis of Suv39h1-/-, Suv39h2-/- double knockout (Suv39h1/h2 dn) MEF cells using the indicated antibodies. DNA was visualized using DAPI. Bars, 5 µm.

Since short peptides do not represent the targeting and binding situation in the cell, we analyzed H3K9me3 binding of ADNP in a chromatin context. We set up an *in vitro* chromatin reconstitution system that makes use of uniformly K9me3 modified H3 obtained by native protein ligation (see Figure S1 and [47], [48], [49], [50] for details of the recombinant chromatin template). To analyze ADNP interaction biotinylated recombinant chromatin with or without the H3K9me3 modification was bound to streptavidin coated beads. This matrix was used to pull out binding proteins of HeLa S3 nuclear extracts. After extensive washing the bound fractions were analyzed by western blotting. As Figure 2A shows, ADNP was clearly enriched on the H3K9me3 chromatin template compared to the unmodified chromatin control or the mock (beads only) reaction.

Next, we analyzed the subcellular localization of ADNP by immunostaining MEF cells using an anti-ADNP antibody. DNA was counterstained with DAPI to visualize heterochromatic areas, which are mainly representing pericentromeric heterochromatin. In mouse cells these areas are generally enriched in the H3K9me3 histone PTM [8], [51]. ADNP staining in interphase cells revealed a speckled distribution in the cell nucleus with additional enrichment at DAPI-dense regions. There, significant overlap with the H3K9me3 modification was observed (Figure 2B). In addition, tubulin-like staining was seen in the cytoplasm of some cell immunofluorescence preparations. A similar distribution was found in NIH3T3 cells at interphase (Figure S3). The tubulin-like staining was also observed in mitotic NIH3T3 cells where we found ADNP excluded from chromatin from metaphase to telophase (Figure S3). In subcellular fractionation experiments of NIH3T3 cells no ADNP could be detected in the cytoplasmic fraction codistributing with β-tubulin. In contrast, ADNP was found largely enriched in the nuclear insoluble chromatin fraction (Figure 2C). As tubulin-like staining of the anti-ADNP antibody also varied with fixation conditions (data not shown), we infer the cytoplasmic patterning of ADNP to be an artifact of the antibody staining procedure.

Due to the large number of histone H3 genes in mammalian cells, the H3K9 site cannot be mutated in this system. Therefore, trimethylation of H3K9 cannot be removed from the cells directly. To address the role of H3K9me3 in the enrichment of ADNP at pericentromeric heterochromatin we repeated the anti-ADNP immunolocalization in Suv39h1, Suv39h2 double knockout cells. Suv39 is the methyltransferase that is mainly responsible for H3K9me2/3 at pericentromeric heterochromatin. In the absence of Suv39h1/h2 H3K9me3 at pericentromeric heterochromatin of mouse cells is lost [51]. Indeed, as Figure 2D shows no enrichment of H3K9me3 at DAPI-dense areas could be seen in the Suv39h1, Suv39h2 double knockout MEF cells. ADNP was indeed found absent from pericentromeric heterochromatin in these cells. In contrast, the speckled distribution of ADNP throughout the nucleus was still visible. From these experiments we conclude that ADNP enrichment at pericentromeric heterochromatin is dependent on the presence of Suv39h1/h2 enzymes and likely is governed by recruitment of the factor to the H3K9me3 chromatin mark.

### ADNP does not bind H3K9me3 directly but is targeted to pericentromeric heterochromatin via HP1

**Figure 3:**
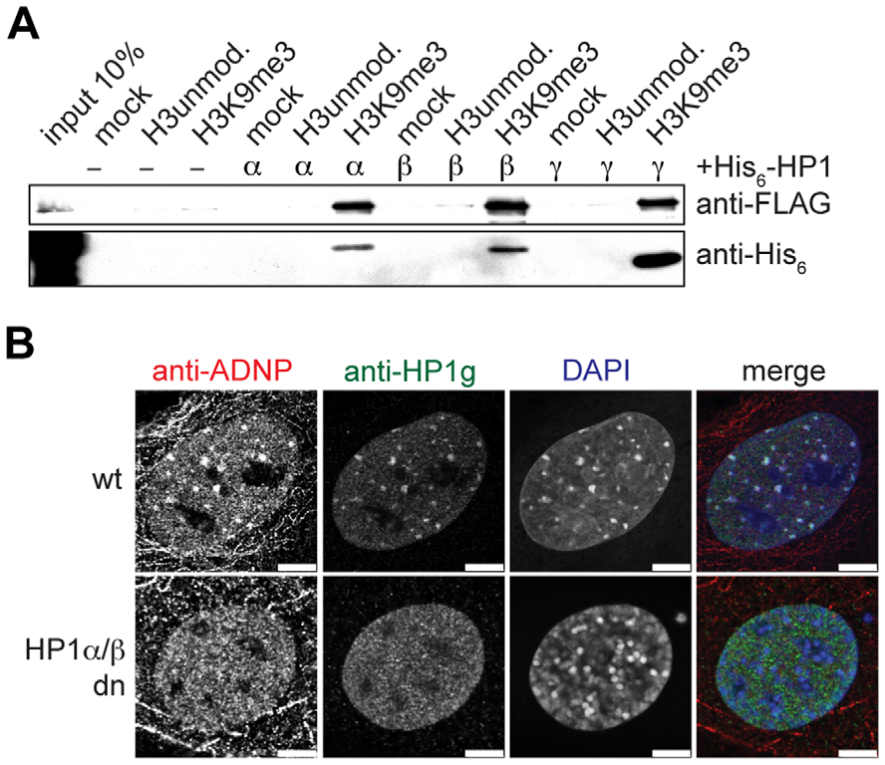
ADNP is recruited to H3K9me3 via HP1. (**A**) The indicated histone H3 peptides were used in pulldown experiments using TNT reticulocyte lysate programmed to express FLAG-tagged ADNP. Beads without coupled peptides were used as control (mock). Where indicated recombinant His_6_-tagged HP1 proteins of the different α, β or γ isoforms were added. Pulldown reactions were analyzed by western blotting using anti-FLAG and anti-His_6_ antibodies. (**B**) Wild type (wt) and HP1α-/-, HP1β-/- double knockout (HP1α/β dn) MEF cells were stained with the monoclonal anti-ADNP and polyclonal anti-HP1γ antibodies. DNA was visualized using DAPI. Bars, 7.5 µm.

ADNP was recently suggested to interact with HP1α [45]. Therefore, we asked whether ADNP is capable of binding to H3K9me3 directly or only in the presence of HP1. We performed peptide pulldown experiments using H3K9me3 and unmodified H3 peptides immobilized on magnetic beads and beads without peptides as control (Figure 3A). FLAG-tagged ADNP was expressed in TNT reticulocyte extract and incubated with the different affinity matrices in the absence or presence of recombinant HP1 isoform proteins. Without HP1 ADNP did not bind to any of the differentially loaded beads with or without immobilized peptides. However, when co-incubated with recombinant HP1α, HP1β or HP1γ ADNP bound specifically to the H3K9me3 peptide. This interaction was concomitant with recruitment of the different HP1 isoform proteins to the H3K9me3 matrix. In contrast, we detected only background interaction of ADNP or the different HP1 proteins to the unmodified peptide.

To test whether recruitment of ADNP to H3K9me3 is also mediated by HP1 *in vivo*, we made use of mouse HP1-/- knockout cells [52], [53], [54]. Immunofluorescence analysis of HP1α, HP1β or HP1γsingle knockout MEF cells showed that ADNP localization to DAPI dense areas of pericentromeric heterochromatin is not affected by the lack of one single HP1 isoform (Figure S4). We then expanded the analysis to HP1αHP1β double knockout MEF cells. Here, HP1γ is the only HP1 isoform expressed. In immunofluorescence analysis of corresponding wild type MEF cells we found HP1γ partially enriched at pericentromeric heterochromatin where it colocalized with DAPI dense areas. A diffuse nuclear staining was also visible (Figure 3B, top row). In contrast, in the HP1αHP1β double knockout MEF cells HP1γ did not display enrichment at DAPI dense areas but only showed nuclear diffuse distribution (Figure 3B, bottom row). Interestingly, absence of HP1α and HP1β did not have a pronounced effect on pericentromeric heterochromatin as DAPI dense areas that coincided with H3K9me3 were still observed (Figure S5). No enrichment of ADNP at DAPI dense areas could be detected in HP1αHP1β double knockout MEF cells (Figure 3B, bottom row). Instead, only punctuate nuclear staining was observed throughout the nucleoplasm. To verify that ADNP displacement from pericentromeric heterochromatin in these cells is not an effect of absence of H3K9me3, or loss of ADNP and/or HP1γ we assessed the protein and modification levels using western blotting. No significant changes in the expression levels of ADNP or HP1γ or the level of H3K9me3 could be detected in HP1αHP1β double knockout MEF cells compared to corresponding wild type MEF cells (Figure S6). We conclude that presence of HP1α or HP1β is necessary for recruitment of ADNP to H3K9me3 containing pericentromeric heterochromatin.

### The homeodomain of ADNP is necessary and sufficient for localization to pericentromeric heterochromatin

**Figure 4:**
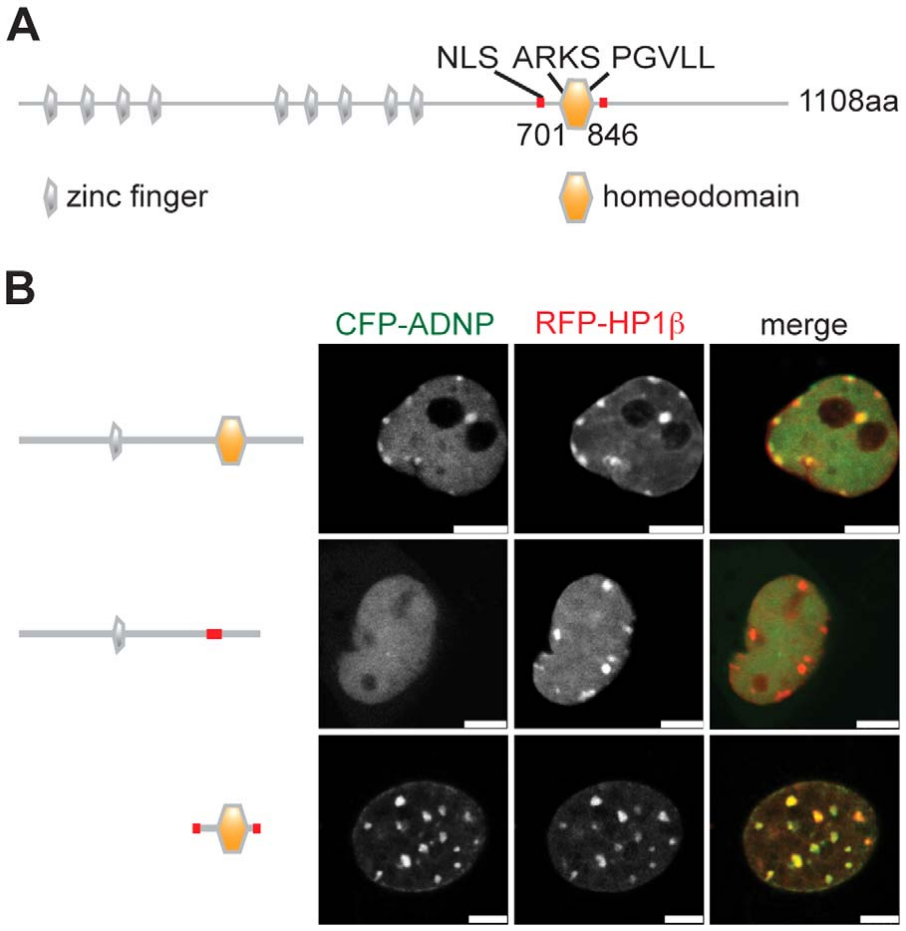
The homeodomain of ADNP is necessary and sufficient for localization to pericentromeric heterochromatin. (**A**) Schematic representation of ADNP protein structure (Uniprot ID Q9H2P0). Positions of the nine zinc-fingers (grey), the homeodomain (orange), the nuclear localization signal (NLS) and the ARKS and PGVLL motifs are indicated. (**B**) NIH3T3 cells were transfected with CFP-ADNP, CFP-ADNP(D741-846) (homeodomain deletion), CFP-ADNP(701-846) (NLS and the homeodomain) and RFP-HP1β. Only cells of low to medium expression levels showed distribution of wt CFP-ADNP similar to the endogenous protein. Therefore, the images show CFP and RFP fluorescence signals of selected unfixed, living cells of low expression levels of the different CFP-fusion proteins using a confocal microscope. Bars, 5 µm.

Besides nine Zn fingers the primary sequence of ADNP contains a predicted NLS as well as a homeodomain (Figure 4A). Within the homeodomain a PGVLL region at position 819–823 could serve as an PxVxL HP1-interaction motif [28]. To test whether the ADNP homeodomain is indeed sufficient for localization of the factor to pericentromeric heterochromatin *in vivo* we analyzed two different deletion mutants of the protein. Shown in Figure 4B are CFP-ADNP constructs corresponding to full-length ADNP, the protein lacking the homeodomain [CFP-ADNP(Δ741-846)] as well as the homeodomain alone [CFP-ADNP(701-846)] all containing the nuclear localization signal (NLS). These constructs were transiently co-expressed with RFP-HP1β as a heterochromatin marker in NIH3T3 cells. Analysis of unfixed, living cells of low expression level of the fusion constructs revealed subnuclear enrichment of the wild type CFP-ADNP fusion protein to pericentromeric heterochromatin reminiscent of the untagged, endogenous factor. Interestingly, the punctuate distribution of the exogenously overexpressed fusion protein was far less pronounced compared to immunodetection of the endogenous protein using the anti-ADNP antibody (compare to Figure 2A). While full-length ADNP and the homeodomain alone co-localized with HP1β the mutant protein lacking the homeodomain did not display enrichment at any particular site in the nucleus. Obviously, the homeodomain is required and sufficient for ADNP localization to pericentromeric heterochromatin.

### Besides a PGVLL sequence patch an ARKS motif within the ADNP homeodomain is involved in HP1 dependent H3K9me3 association and localization to pericentromeric heterochromatin

**Figure 5:**
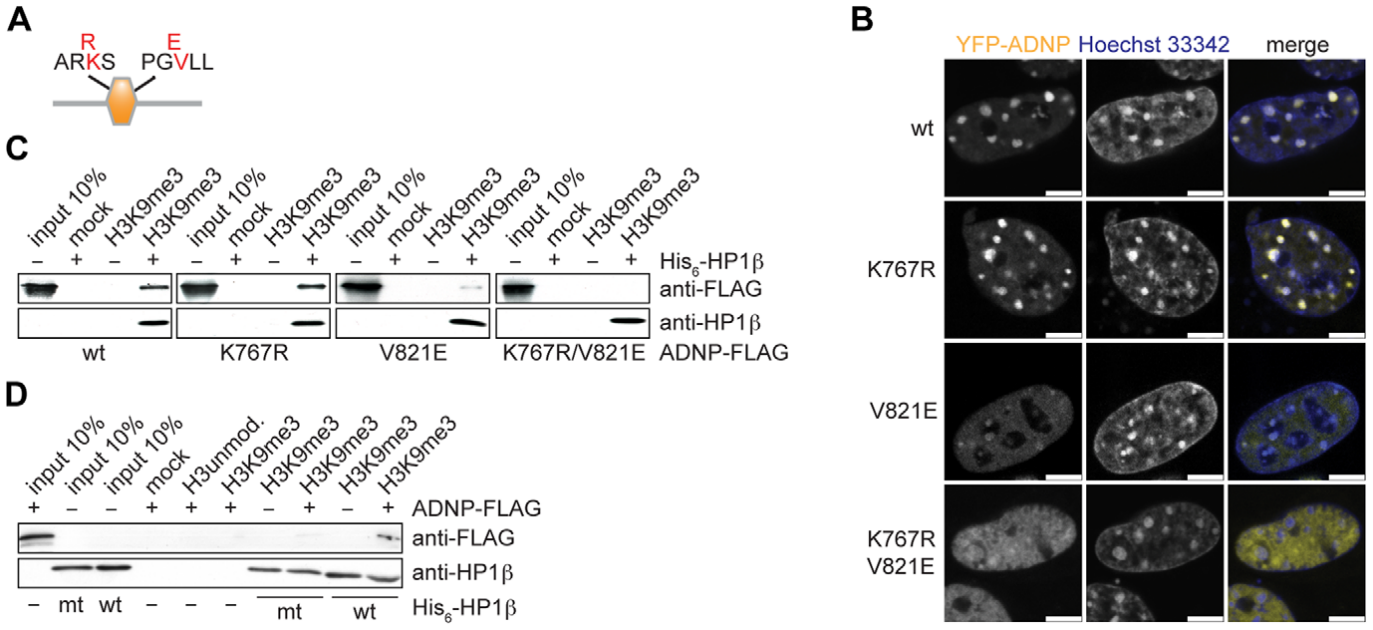
PGVLL and ARKS motifs within the ADNP homeodomain are involved in HP1 chromoshadow domain interaction. (**A**) Schematic representation of the ADNP-homeodomain (region aa 701–846), containing a K to R mutation in the ARKS and a V to E mutation in the PGVLL motif. (**B**) Fluorescence analysis of unfixed, living NIH3T3 cells inducibly expressing the indicated wild type (wt) and point mutant YFP-ADNP fusion proteins. DNA was stained with Hoechst 33342 dye. Bars, 5 µm. (**C**) Pulldown experiment with H3K9me3 peptides immobilized on magnetic beads using TNT-reticulocyte-extract expressed ADNP-FLAG (wt and point mutants) and recombinant wild type His_6_-HP1**β**. Western blot analysis of the reactions using the indicated antibodies is shown. (**D**) Pulldown experiment with H3K9me3 peptides immobilized on magnetic beads using wild type ADNP-FLAG protein expressed in TNT reticulocyte extract expressed together with recombinant wild type (wt) and W170A mutant (mt) His_6_-HP1β. Western blot analysis of the reactions using the indicated antibodies is shown.

Next to a PxVxL HP1 consensus binding PGVLL sequence the ADNP homeodomain contains an ARKS sequence stretch (pos 765–768) (Figure 5A). Within histone H3 methylated ARKS motifs around lysine 9 and lysine 27 have been shown to bind HP1 and Polycomb chromo domain proteins [21]. To further characterize the ADNP–-HP1 interaction, the homeodomain was mutated at the putative HP1 interaction sites. Lysine 767 within the ARKS motif was changed to arginine. This should prevent a possible methylation event, thereby destroying the ARKS binding cassette, which might be bound by the HP1 chromo domain. In the PGVLL motif valine 821 was mutated to a glutamate. A corresponding mutation has been shown to strongly reduce binding to the HP1 chromoshadow domain of Sp100A and LBR [55]. Wild type ADNP, the single point mutants and the double mutant were fused to YFP and stably transfected in NIH3T3 cells using an inducible system. Expression levels of the ADNP wild type and mutant YFP-fusion proteins after induction were comparable, but significantly above the endogenous factor (Figure S7). As with transiently overexpressing cells analysis of unfixed, cells revealed enrichment of wild type YFP-ADNP at DNA-dense areas, which were stained with Hoechst 33342 dye. Also, almost no punctuate distribution in the nucleus was seen. In this setting the K767R mutation had no effect on ADNP localization (Figure 5B). In contrast, the V821E mutation within the PGVLL motif lead to strong reduction of the ADNP signal at regions of pericentromeric heterochromatin. However, in all cells analyzed a faint but significant signal of the mutant YFP-fusion protein remained at pericentromeric heterochromatin. This residual ADNP enrichment was, however, absent for the K767K/V821E double mutant protein.

We then confirmed these results *in vitro*. Wild type and mutant FLAG-tagged ADNP proteins were expressed in TNT reticulocyte extract and used in H3K9me3 peptide pulldown experiments in conjunction with recombinant HP1β. As Figure 5C shows, wild type and K767R mutant ADNP were bound to H3K9me3 peptide in a HP1β dependent manner. In contrast, recovery of the V821E mutant ADNP protein was clearly reduced. No interaction with the double mutant K767R/V821E ADNP protein was observed under the same conditions.

If interaction between the HP1 chromoshadow domain and the PGVLL motif is the main mechanism of ADNP binding to H3K9me3, then association of ADNP with H3K9me3 should be strongly reduced by a mutation within this region. Mutation of tryptophan 170 to alanine (W170A) of HP1β has been described to abolish HP1-PxVxL interaction without affecting HP1 dimerization and H3K9me3 binding [27]. We analyzed this recombinant, mutant HP1βprotein in H3K9me3 peptide pulldown experiments with wild type FLAG-tagged ADNP expressed in TNT reticulocyte extract (Figure 5D). W170A mutation had no effect on HP1β binding to the H3K9me3 peptide. Yet, recruitment of ADNP to H3K9me3 was severely impaired in presence of the mutant HP1β W170A protein compared to the wild type HP1β protein. We further note that ADNP did have no effect on the recruitment of HP1β to the H3K9me3 peptide in these experiments. We conclude that interaction of the PGVLL motif within the homoeodomain with the chromoshadow domain dimer of HP1 is a major element of ADNP H3K9me3 association. However, there is contribution, albeit minor, of the ARKS motif that also affects H3K9me3 association and localization of ADNP to heterochromatin.

### ADNP functions in silencing of major satellite repeats

**Figure 6:**
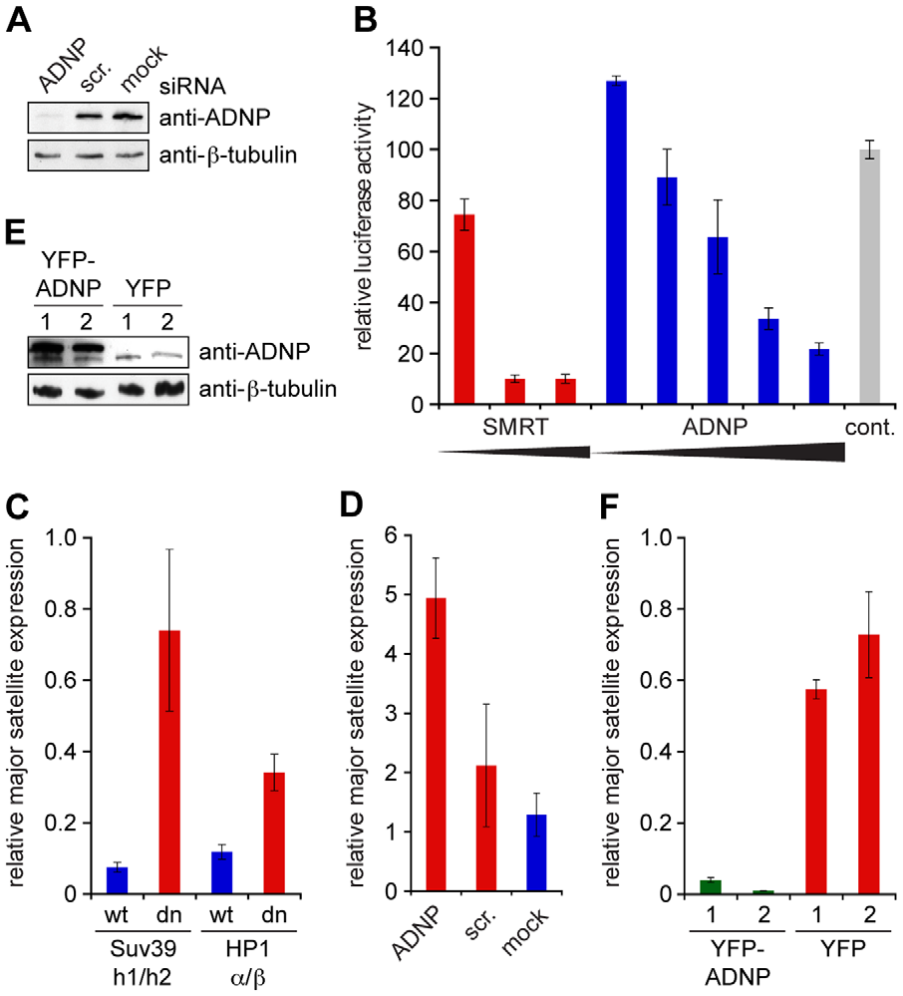
ADNP represses major satellite repeat transcription. (**A**) NIH3T3 cells were transfected with siRNA against ADNP. As controls NIH3T3 cells transfected with a corresponding scrambled siRNA (scr.) and untransfected cells (mock) were used. Western blot analysis using anti-ADNP and anti β-tubulin (loading control) antibodies is shown. (**B**) Increasing concentrations of GAL4-SMRT (positive control) or GAL4-ADNP expressing plasmids were transfected into 293HEK TK22 cells which contain a luciferase reporter downstream of a UAS and a minimal TK promotor integrated into the genome. pcDNA3.1 plasmid was used as control. Luciferase activity normalized to transfection efficiency determined by expression from a renilla luciferase control plasmid is reported. Averages and standard deviations from three independent experiments are given. (**C**) Relative amounts of transcripts from major satellite repeats in Suv39h1-/-,Suv39h2-/- double knockout (Suv39h1/h2 dn) as well as HP1α-/-,HP1β-/- double knockout (HP1α/β dn) MEF cells and corresponding wild type (wt) MEF cells were determined relative to the levels of GAPDH mRNA using reverse transcription and real time PCR. Average 2^-Δct^ values and standard deviation (error bars) of a triplicate experiment is blotted. (**D**) Relative levels of transcripts from major satellite repeats in NIH3T3 cells treated with ADNP siRNA, corresponding scrambled siRNA (scr.) or untransfected cells (mock) as analyzed in (C). (**E**) Western blot analysis of NIH3T3 cells after transfection with YFP-ADNP or YFP expressing plasmids using the indicated antibodies. Results of two independent experiments (1, 2) are blotted. (**F**) Relative levels of transcripts from major satellite repeats in stably transfected NIH3T3 cells after transient induction of YFP-ADNP or YFP expression as analyzed in (C). Result of two independent experiments (1, 2) are blotted.

Misregulation of chromatin modification marks, delocalization of HP1 and derepression of major satellite repeat transcription have been described in Suv39h1,Suv39h2 double knockout MEF cells [16], [56]. Since ADNP is lost from pericentromeric heterochromatin in these cells (see Figure 2), we reasoned that ADNP might act downstream of Suv39 and H3K9me3 in maintenance pathways of constitutive heterochromatin. To assess ADNP function at pericentromeric heterochromatin we established siRNA knockdown in NIH3T3 cells. As Figure 6A shows, transfection of ADNP siRNA lead to significant reduction of protein levels compared to the scrambled siRNA and untransfected (mock) controls. First, we assessed possible effects of reduced ADNP amounts onto histone methylation marks associated with pericentromeric heterochromatin. However, no effects on the levels and distribution of H3K9me3, H3K27me1 and H4K20me3 could be observed in ADNP knockdown NIH3T3 cells (Figure S8 and S9). Similarly, no reduction of CpG methylation at major satellite repeat sequences could be detected in these cells (Figure S10). Also, distribution of the HP1α, HP1β and HP1γ proteins appeared normal after ADNP knockdown (Figure S11).

ADNP has been demonstrated to be involved in transcriptional activation as well as repression during mouse embryogenesis [45]. To analyze whether ADNP plays a role in Suv39h1/h2 directed silencing of major satellite repeats [16] we first addressed the transcriptional activating or repressive potential of ADNP. In chromatin context we found a GAL4(DBD)-ADNP fusion protein when targeted to an upstream UAS to slightly activate transcription at low doses, but to repress basal transcription at higher doses from a minimal TK promoter construct, which was stably integrated into the genome of 293 HEK cells (Figure 6B). The repressive activity observed for ADNP was not as high as for SMRT a well-established transcriptional corepressor [57], but nevertheless robust. Similar results were observed when the reporter construct was not stably integrated, but transiently transfected in 293 HEK cells (data not shown).

To test whether ADNP has silencing function at pericentromeric heterochromatin we determined the transcriptional status of major satellite repeats. We measured the abundance of major satellite transcripts relative to GAPDH using reverse transcription and real time PCR. In such analysis Suv39h1,Suv39h2 as well as HP1αHP1β double knockout MEF cells showed clear increase in major satellite repeat transcription compared to the respective wild type MEF cells (Figure 6C). Interestingly, in the ADNP knockdown NIH3T3 cells the level of major satellite transcripts was also elevated compared to the cells treated with the scrambled control siRNA or untransfected (mock) cells (Figure 6D). If ADNP is causally involved in silencing of major satellite repeat transcription, then increased ADNP levels should result in further repression. To test this hypothesis, we overexpressed YFP-ADNP in NIH3T3 cells. As Figure 6F shows elevated ADNP levels indeed caused significant decrease of transcripts from the major satellite repeats. We conclude that ADNP is causally involved in silencing of these essential repetitive DNA elements.

## Discussion

### ADNP localization to H3K9me3 marked pericentromeric heterochromatin

Until now ADNP studies have focused on extracellular signaling functions in neuroprotection [37], [44], [58], [59], [60], [61] or transcriptional regulation of individual genes in euchromatin [42], [45]. And while biochemical studies have found association of ADNP with components of the SWI/SNF chromatin remodeling complex [46], we describe here a novel aspect of ADNP biology, accumulation at pericentromeric heterochromatin. We ascribe the fact that ADNP has not been seen at these pronounced subnuclear structures before mainly to two effects. Immunofluorescence analysis with anti-ADNP antibodies requires special fixation and staining techniques. Otherwise no significant enrichment at pericentromeric heterochromatin can be observed. Also, in different cell systems (e.g. mouse vs. human cells and fibroblasts vs. neuronal cells) the overall localization of ADNP might vary. We are confident that ADNP is a bona fide heterochromatin accessory protein as imaging of live cells stably expressing YFP-ADNP in the absence of any fixation procedure also shows strong enrichment of the protein at DAPI-dense regions (Figure 5B). We do not think that this is an effect of overexpressing ADNP as we biochemically observed clear enrichment of endogenous ADNP at H3K9me3 a hallmark of heterochromatin in the context of histone peptides as well as recombinant chromatin. Our fractionation results in combination with the stable transfected cell lines indicate tubulin-like patterning of ADNP immunostaining in the cytoplasm is artificial. Lastly, we think that dotted appearance of ADNP immunostaining in the cell nucleus might be a reflection of the staining procedure as almost no such distribution was detected in the YFP-ADNP cell lines without fixation.

### Role of HP1 in ADNP localization

While we identified ADNP in a biochemical screen for H3K9me3 interacting factors, subsequent experiments indicated ADNP does not directly bind this histone PTM. Analysis of the ADNP primary sequence does not reveal any of the characterized methyl-lysine binding modules such as chromo domains, PHD fingers or ankyrin repeats [62], [63]. Indeed, *in vitro* translated ADNP from reticulocyte extracts does not interact with H3K9me3 peptides. Conversely, we find that all three mammalian HP1 isoforms, HP1α, HP1β and HP1γ bind to ADNP and mediate its interaction with H3K9me3 *in vitro*. Further, we observed exclusion of ADNP from chromosomes during M-phase (Figure S3) simultaneously to HP1, which indicates HP1 dependent chromatin recruitment [64]. While knockout of single HP1 isoforms did not affect ADNP localization, the protein was absent from pericentromeric heterochromatin in HP1αHP1β double knockout MEF cells possibly indicating that targeting of ADNP can be mediated by HP1α or HP1β but not by HP1γ Obviously, heterodimerization of HP1γ with HP1α or HP1β or factors regulated by HP1α and HP1β are necessary for HP1γ localization to pericentromeric heterochromatin [53]. We do not yet know whether HP1α or HP1β also require heterodimerization with other isoforms for localization or whether they act self-sufficient in ADNP recruitment. Further studies will also have to address whether HP1γ is at all capable of interacting with ADNP in cells. In this context it is nevertheless noteworthy that HP1β animals as well as siRNA studies involving HP1α “knock-down” indicate neuronal defects [52], [65]. Disruption of ADNP–HP1αβ interaction and proper ADNP localization might here be causally involved.

Apart from HP1 proteins additional factors could contribute to ADNP heterochromatin localization. For example, Brg1 of the SWI/SNF complex interacts with the C-terminus of ADNP [46]. Brg1 knock-out causes misorganization of pericentromeric heterochromatin [66]. Further, potential binding of ADNP to DNA could stabilize heterochromatin association. While ChIP studies have shown that ADNP can bind multiple gene promoters [45] it is unclear whether this interaction is direct. With the large number of putative DNA binding domains (nine Zn fingers, homeodomain) ADNP could possibly be attracted to repetitive DNA sequences within heterochromatin.

### Protein motifs involved in ADNP localization

Our results indicate that the CSD of HP1 proteins mediate ADNP interaction, which is reminiscent of several HP1 protein interactions that have been analyzed [67], [68]. However, we show that besides a CSD interacting PxVxL consensus PGVLL motif an ARKS motif is also involved in H3K9me3 association and ADNP localization. We consider four scenarios. (i) The ARKS motif might influence ADNP multimerization, which might be required for stable HP1 binding. We exclude this possibility, since all ectopically expressed ADNP homeodomain mutant proteins interact with the endogenous protein comparable to the wild type factor (Figure S12). (ii) The lysine residue within the ARKS motif might be methylated *in vivo* thereby generating a HP1 CD interaction interface. Synergistic binding of the HP1 CD to this PTM and the CSD to the PGVLL motif might be required for stable ADNP targeting. HP1 CSD dimerization might allow for simultaneous HP1 CD binding to H3K9me3 and to the ADNP ARKS motif. So far, we did not detect ADNP methylation in *in vivo* or *in vitro* labeling experiments. Also, interaction of recombinant and thereby unmethylated ADNP with HP1 is significantly reduced after K767R mutation, an effect that recuperates the *in vivo* findings. Lastly, we directly tested HP1 interaction with a methylated peptide spanning the ADNP ARKS region and did not detect binding (Figure S13). (iii) The ARKS motif might be required for homeodomain full structural integrity. Since HP1 has been shown to bind several proteins within different domains via PxVxL motifs this type of interaction seems not to require a certain structure. (iv) The ARKS motif might bind to another factor that stabilizes HP1 ADNP interaction and localization to pericentromeric heterochromatin.

### ADNP function at pericentromeric heterochromatin

Complete deficiency of ADNP in mouse embryos results in changes in gene expression profile [45]. However, it is unclear whether ADNP is directly or indirectly involved in euchromatin gene regulation. We show transcriptional repressive activity of ADNP when targeted to a reporter gene. Our results of ADNP knockdown indicate derepression of major satellite repeat transcription. We do not detect any effect of reduced ADNP levels onto HP1 localization, heterochromatic histone PTM levels and localization, nor onto DNA methylation levels. These are all effects that have been observed in Suv39h1, Suv39h2 double knockout cells [9], [16], [34], [51], [56]. As we show ADNP is downstream of the Suv39/H3K9me3/HP1 axis, we think that ADNP has a more direct effect onto transcription at major satellite repeats. How could ADNP exert this effect? Interaction between ADNP and three members of the SWI/SNF chromatin-remodeling complex (BAF250a, BAF170 and Brg1) has been shown by co-immunoprecipitation [46]. Although Brg1 is not enriched at DAPI dense regions, its deficiency results in dissolution of pericentromeric heterochromatin and delocalization of the heterochromatin markers H3K9me3 and H4K20me3 [66]. ADNP could be involved in that process by recruiting Brg1 (perhaps transiently) or by modulating the activity of the SWI/SNF complex. Alternatively, ADNP might directly target RNA polymerase(s) or basal transcription factors involved in major satellite transcription.

## Materials and Methods

### DNA Constructs

For protein expression cDNA corresponding to the open reading frame of ADNP was amplified from an EST clone (IMAGE: 3448801, GenBank NM_009628) using PCR. For *in vitro* experiments ADNP and point mutants were cloned into a derivative pcDNA3.1 vector (Invitrogen) generating C-terminal fusion to a 2x FLAG-2xHA epitope tag. For expression in mammalian cells the cDNA was cloned into pECFP-C1, pEYFP-C1 (Clontech) and DBD-GAL4-null (gift of Dr. J. Rice, [69]) and subcloned from pEYFP-C1 into pGeneV5 (Invitrogen). Mutagenesis (K767R, V821A and the homeodomain deletion Δ741-846) was performed by fusion PCR. The homeodomain was subcloned by PCR using primers for the region encoding aa 701-846. Human HP1α, HP1β and HP1γ (GenBank: BC006821, BC002609 and BC000954) directly fused to a His_6_-tag encoding sequence (5′-ATGAAAAAACACCACCACCACCACCAC-3′) at the 5′-end was cloned into pET11a vector (Novagen). The CMV renilla and Gal4-SMRT plasmids were gifts of Dr. J. Rice [69] and the mRFP-HP1β plasmid was obtained from Dr. B. van der Zaal. Further details of the used DNA constructs are available upon request.

### Southern Blot Analysis

Genomic DNA was prepared as described [70] and digested with TaiI restriction enzyme (NEB). Samples were separated on 1% agarose gels and stained with ethidium bromide. Southern blot was performed according to [70] with modifications: Gels were rinsed in H_2_O, depurinated for 20 min in 0.25 M HCl and neutralized for 30 min in 0.4 M NaOH. Downward transfer of the DNA onto a positively charged nylon transfer membrane (GE Healthcare) was performed over night using 0.4 M NaOH as transfer buffer. Major satellite DNA was detected using the DIG High Prime DNA Labeling and Detection Starter Kit I (Roche Diagnostics). The probe for major satellite was generated by PCR using DIG-labeled dUTP: dTTP in a ratio of 1: 3. Genomic DNA isolated from NIH3T3 cells served as template. Major satellite repeat primers were Fwd: 5′-ATATGTTGAGAAAACTGAAAATCACG-3′ and Rev: 5′-CCTTCAGTGTGCATTTCTCATTTTTCAC-3′ [71].

### Real Time PCR

Total RNA was extracted using TRIzol® reagent (Invitrogen). First strand synthesis of cDNA was performed using the Superscript II Kit (Invitrogen). Before cDNA synthesis contaminating genomic DNA was removed by digestion with DNAse I (1 U/µg RNA, 30 min, 37°C) (NEB). Reactions were stopped by adding EDTA to a final concentration of 10 mM and heating for 10 min at 75°C. cDNA was used for real time PCR using IQ SYBR Green Supermix on a MJ Research DNA engine Opticon (Bio-Rad). To calculate the relative expression levels, the major satellite signals were normalized to the signals of GAPDH as follows: Ct_major satellite repeat_ – Ct_GAPDH_  =  ΔCt. Averages of the 2^-ΔCt^ values from triplicate experiments were blotted with the standard deviation of the triplicates as error bars. The PCR primers amplifying transcripts from major satellite repeats were the same as for generating the Southern blot probe. GAPDH primers: Fwd: 5′-AGGTCGGTGTGAACGGATTTG-3′, Rev: 5′-TGTAGACCATGTAGTTGAGGTCA-3′.

### Western Blotting

For western blot analysis primary antibodies were used as follows: anti-ADNP (BD Biosciences), 1∶1,000; anti-H4 (Abcam), 1∶1,000; anti-β-tubulin (Sigma), 1∶20,000; anti-FLAG (Sigma), 1∶1,000; anti-His_6_ (Santa Cruz), 1∶250.

### Peptide and Chromatin Pulldowns

Peptides used for pulldown studies carried a biotinylated lysine residue at the C-terminus: H3unmod., ARTKQTARKSTGGKAPRKQLK-biotin; H3K9me3, ARTKQTARK(me3)STGGKAPRKQLK-biotin. Recombinant chromatin carrying the same modifications was prepared as described [47]. Peptide and chromatin pulldowns were carried out according to [47]. Proteins were either detected by western blot procedures or analyzed by mass spectrometry (MS).

### Mass Spectrometry

SDS PAGE gels were stained with Coomassie Blue and entire gel lanes were cut into 23 slices of equal size. Proteins within the slices were digested according to Shevchenko et al. [72]. Peptides were extracted and analyzed by LC-coupled tandem MS on an Orbitrap Xl mass spectrometer (Thermo Fisher Scientific). CID fragment spectra were searched against NCBInr database using MASCOT as search engine.

### Cell Culture

NIH3T3 (ATCC) and HEK 293 TK22 (gift of Dr. J. Rice, [69]) cells were grown at 37°C in a humidified atmosphere, 5% CO_2_ using Dulbecco's modified Eagle's medium supplemented with 10% fetal bovine serum, 2 mM glutamine, and 1x penicillin/streptomycin (Invitrogen) (DMEM+). Suv39h1, Suv39h2 double knockout and wild type MEF cells (gift of Dr. T. Jenuwein) [51] and HP1α HP1β HP1γ or HP1αHP1β double knockout and wild type MEF cells [52], [53], [54] were grown at 37°C in a humidified atmosphere, 5% CO2 using DMEM+ supplemented with 1x non essential amino acids, 1x sodium pyruvate.

### ADNP Expressing Cell Lines

One day prior to transfection GeneSwitch NIH3T3 cells (Invitrogen) were seeded in 10 cm dishes to reach ∼50% confluency at the day of transfection. Typically 16 µl JetPEI (Biomol) reagent and 8 µg of YFP or YFP-ADNP plasmid were used for transfection per dish. One day after transfection, cells were trypsinized and transferred to 15 cm dishes containing medium supplemented with 400 µg/ml zeocin (Invitrogen). After separate colonies were visible, expression of YFP/YFP-ADNP was induced with 10 ng/ml Mifepristone (Sigma). Cells were washed with PBS and covered with 37°C warm 0.5% low melting agarose in PBS. YFP/YFP-ADNP expressing cells were picked using cloning cylinders and transferred into 24 well plates. Stable inducible colonies were analyzed by fluorescence microscopy (Axiovert 40 CFL, Zeiss) and western blotting. Two colonies of moderately overexpressing cell-lines for each construct were frozen for long-term use and storage.

### RNAi

NIH3T3 cells were seeded in 6-well plates one day prior to transfection to reach 30-50% confluency at the day of transfection. 100 µg siRNA was transfected with 5 µl Lipofectamine2000 as described by the manufacturer (Invitrogen). The following si RNARNAs were used: ADNP-siRNA, sense 5′ - [GAUUCUUAUGAGGCUAGGA] RNA [TT] DNA, antisense 5′ - [UCCUAGCCUCAUAAGAAUC] RNA [TT] DNA; scrambled siRNA, sense 5′ - [AGGUAGUGUAAUCGCCUUG] RNA [TT] DNA, antisense 5′ - [CAAGGCGAUUACACUACCU] RNA [TT] DNA (MWG). One day post transfection cells were trypsinized and transferred to 10 cm dishes where transfection was repeated with 600 µg siRNA and 30 µl Lipofectamine2000. The next day cells were washed with ice cold PBS, scraped off the plates and aliquoted for protein, DNA and RNA extraction.

### Luciferase Reporter Assay

HEK293 TK22 cells were seeded at a density of 1.5×10^5^ cells/well into 12-well plates 24 h prior treatment. A total amount of 1.2 µg DNA per well was transfected using Lipofectamine2000 (Invitrogen). For transfection the total DNA contained 2 ng of CMV renilla plasmid, the plasmids expressing the GAL4-tagged protein of interest in different amounts (10 ng–200 ng) and empty pcDNA3.1 vector to reach 1.2 µg plasmid DNA in total. 48 h post transfection cells were detached from the wells by pipetting in medium and transferred to 1.5 ml Eppendorf tubes. Cells were pelleted by centrifugation for 5 min at 3000 rpm, RT. Cell pellets were lysed and processed for dual luciferase assay (Promega) according to the manufacturer's protocol. Firefly and renilla luminescence signals were read using a Chameleon II plate reader (Hidex Oy). Firefly luminescence signals were normalized against renilla luminescence signals and control transfected cells.

### Immunofluorescence

Cells were seeded on glass cover slips in 6-well plates. For ADNP staining in interphase cells were treated with nocodazole (200 nM, in DMSO) for 5 min prior to fixation. Cover slips were washed two times with PBS and fixed for 10 min in 3% paraformaldehyde in PBS at 37°C. Cover slips were washed once with PBS and cells were permeabilized for 7 min (0.2% Triton X-100 in PBS). Cells were washed once with PBS and blocked for 30 min in blocking solution (1x PBS, 2% BSA, 5% normal goat serum (v/v)). Primary antibodies (anti-ADNP (BD Biosciences) 1∶70; anti-H3K9me3 (Millipore) 1∶1,000; anti-HP1α (Millipore) 1∶5,000; anti-HP1β (Millipore) 1∶10,000; monoclonal anti-HP1γ (Millipore) 1∶15,000; polyclonal anti-HP1α, anti-HP1β and anti-HP1γ (Millipore) 1∶1,000) and fluorescently labeled secondary antibodies (anti-mouse-Alexa555 and anti-rabbit-Alexa488 (Molecular Probes) 1∶1,000) were applied in blocking solution for 1 h at room temperature followed by a post fixation step (3% paraformaldehyde in PBS, 15 min at RT). Cover slips were washed three times in water and mounted in Mowiol including 50 µg/ml DAPI. Slides were dried overnight at RT and analyzed using a Leica TCS SP5 confocal microscope. For life cell imaging cells were grown in glass bottom dishes in DMEM+ lacking phenol red.

### ADNP and histone peptides

Peptides used for pulldown experiments were as follows: ADNPunmod. biotin-EDDSYEARKSFLTKYFNKQP; ADNPK767me3, biotin-EDDSYEARK(me3)SFLTKYFNKQP; H3unmod., ARTKQTARKSTGGKAPRKQLK-biotin; H3K9me3, ARTKQTARK(me3)STGGKAPRKQLK-biotin. Peptides used for FP measurements were as follows: ADNPunmod., EDDSYEARKSFLTKY; ADNPK767me3, EDDSYEARK(me3)SFLTKY; H3unmod., ARTKQTARKSTGGKA; H3K9me3, ARTKQTARK(me3)STGGKA.

### Fluorescence Polarization Binding Assays

Peptides used for fluorescence polarization (FP) measurements were labeled using NHS-fluorescein (Invitrogen). Single labeled species were separated by C18 reversed phase HPLC and analyzed by MALDI MS. FP assays were essentially carried out and analyzed as described [73]. Titration series of 10 µl volume in 384 well plates were read multiple times on a Plate Chameleon II plate reader (HIDEX Oy). Multiple readings and independent titration series were averaged after data normalization.

## Supporting Information

Figure S1 **Characterization of recombinant chromatin templates.** (**A**) Agarose gel (1%) of free DNA template (12×200-601 DNA) and the indicated chromatin assembly reactions using either unmodified H3 or H3K9me3 stained with ethidium bromide. The running position of size standards (MW) is indicated on the left. (**B**) The indicated free DNA and chromatin assembled chromatin templates were digested with Mnase for the indicated time periods. Reactions were run on a 1% agarose gel and stained with ethidium bromide. (**C**) The indicated chromatin templates were run on an SDS PAGE gel and stained with Coomassie Blue. The running position of size standards (MW) is indicated on the left. (**D**) H3unmod. and H3K9me3 chromatin templates were analyzed by western blotting using the indicated antibodies.
(TIF)

Figure S2 **Specificity of the anti-ADNP monoclonal antibody.** Extracts prepared from NIH3T3 cells treated with ADNP siRNA, corresponding scrambled siRNA (scr.) or untransfected (mock) were analyzed by western blotting using anti-ADNP monoclonal antibody or anti-β-tubulin antibodies. Different exposures of the ECL secondary antibody detection reaction are shown. Black arrowhead marks running position of ADNP; open arrowhead marks running position of β-tubulin.
(TIF)

Figure S3 **Localization of ADNP during the cell cycle.** Immunofluorescence analysis of ADNP in NIH3T3 cells at interphase (**A**) and during M-phase (**B**). DNA was visualized using DAPI. Bars, 5 µm (A and B, lane 2) or 10 µm (B, rows 1, 3–4).
(TIF)

Figure S4 **Absence of single HP1 isoform proteins does not affect ADNP localization to pericentromeric heterochromatin.** Immunofluorescence analysis of ADNP in wild type (wt) or mutant MEF cells of the indicated genomic background after knock out of the indicated HP1 genes. DNA was visualized using DAPI. Bars, 7.5 µm.
(TIF)

Figure S5 **Absence of HP1α and HP1β does not affect localization of H3K9me3.** Immunofluorescence analysis of H3K9me3 in MEF cells derived from HP1αHP1β double knockout mice (HP1αβdn). DNA was visualized using DAPI. Bars, 7.5 µm.
(TIF)

Figure S6 **Absence of HP1α and HP1β does not affect ADNP, HP1γ or H3K9me3 levels.** Western blot analysis of total cell extracts from wild type (wt) and HP1αHP1β double knockout (HP1αβdn) MEF cells using the indicated antibodies.
(TIF)

Figure S7 **Expression levels of YFP-ADNP stable transfected cell lines.** Western blot analysis of untransfected NIH3T3 cells or NIH3T3 cells stably expressing wild type YFP-ADNP (wt) or the indicated single or double mutant fusion proteins using the anti-ADNP antibody. The black arrowhead indicates the running position of endogenous ADNP; the open arrowhead indicates the running position of the YFP-ADNP fusion proteins.
(TIF)

Figure S8 **Nuclear distribution of H3K9me3, H3K27me1 and H4K20me3 is not affected by ADNP knockdown.** Immunofluorescence analysis of H3K9me3 (**A**), H3K27me1 (**B**) and H4K20me3 (**C**) in wild type (wt) and Suv39h1,Suv39h2 double knockout (Suv39h1/h2 dn) MEF cells (top). Immunofluorescence analysis of H3K9me3 (**A**), H3K27me1 (**B**) and H4K20me3 (**C**) in untreated (mock) and NIH3T3 cells transfected with scrambled or ADNP targeting siRNAs (bottom). DNA was visualized using DAPI. Bars, 25 µm (B, upper row) or 5 µm.
(TIF)

Figure S9 **Levels of histone modifications in ADNP knockdown NIH3T3 cells are not changed.** Western blot analysis of untreated (mock) and NIH3T3 cells transfected with scrambled (scr.) or ADNP targeting siRNAs as well as wild type (wt) and Suv39h1, Suv39h2 double knockout (Suv39h1/h2 dn) MEF cells using the indicated antibodies.
(TIF)

Figure S10 **Knockdown of ADNP does not affect DNA methylation levels.** Genomic DNA of untreated (mock) and NIH3T3 cells transfected with scrambled (scr.) or ADNP targeting siRNAs as well as wild type (wt) and Suv39h1, Suv39h2 double knockout (Suv39h1/h2 dn) MEF cells was digested with the restriction enzyme TaiI and separated on a 1% agarose gel. Ethidium bromide staining of the gel (**A**) and Southern blot (**B**) using a major satellite repeat probe are shown.
(TIF)

Figure S11 **Nuclear distribution of HP1α, HP1β and HP1γ is not affected by ADNP knockdown.** Immunofluorescence analysis of HP1α (**A**), HP1β (**B**) and HP1γ (**C**) in wild type (wt) and Suv39h1, Suv39h2 double knockout (Suv39h1/h2 dn) MEF cells (top). Immunofluorescence analysis of HP1α (**A**), HP1β (**B**) and HP1γ (**C**) in untreated (mock) and NIH3T3 cells transfected with scrambled (scr.) or ADNP targeting siRNAs (bottom). DNA was visualized using DAPI. Bars, 5 µm.
(TIF)

Figure S12 **Mutation of the ADNP homeodomain does not affect protein multimerization.** The indicated YFP-ADNP fusion proteins or YFP were immunoprecipitated from nuclear extracts of the corresponding stable transfected NIH3T3 cell lines using anti-GFP-antibodies. Western blot analysis of the immunoprecipitated (precipitated) material using the anti-ADNP antibody is shown. The black arrowhead indicates the running position of endogenous ADNP; the open arrowhead indicates the running position of the YFP-ADNP fusion proteins.
(TIF)

Figure S13 **HP1 does not bind to ADNP K767me3.** (**A**) The indicated ADNP and histone H3 peptides were used in pulldown experiments of HeLa S3 cell nuclear extract. Beads without coupled peptides were used as control (mock). Specifically recovered proteins were analyzed by western blotting using the indicated antibodies. (**B**) Fluorescence polarization binding assay of recombinant HP1β using the indicated H3 (unmodified, K9me3) and ADNP (unmodified, K767me3) peptides. The averaged fluorescence polarization signal from three independent titration reactions is shown.
(TIF)
