# Supplementary figures and images for: HP1 Recruits Activity-Dependent Neuroprotective Protein to H3K9me3 Marked Pericentromeric Heterochromatin for Silencing of Major Satellite Repeats
Source: PLoS One. 2011 Jan 18;6(1):e15894. doi: 10.1371/journal.pone.0015894 (PMC3022755; doi:10.1371/journal.pone.0015894)

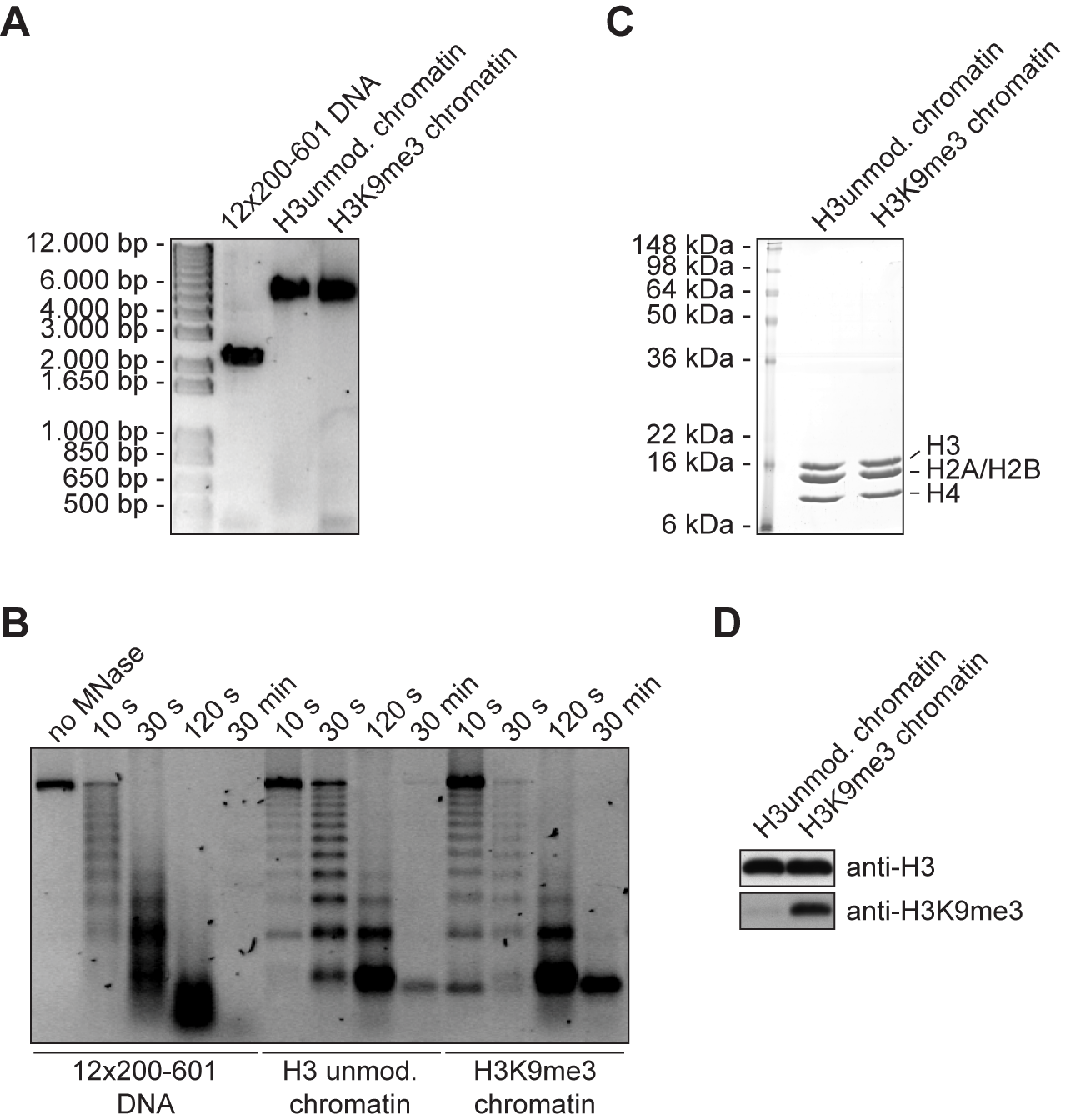

Supplement: Figure S1 — Characterization of recombinant chromatin templates. (A) Agarose gel (1%) of free DNA template (12×200-601 DNA) and the indicated chromatin assembly reactions using either unmodified H3 or H3K9me3 stained with ethidium bromide. The running position of size standards (MW) is indicated on the left. (B) The indicated free DNA and chromatin assembled chromatin templates were digested with Mnase for the indicated time periods. Reactions were run on a 1% agarose gel and stained with ethidium bromide. (C) The indicated chromatin templates were run on an SDS PAGE gel and stained with Coomassie Blue. The running position of size standards (MW) is indicated on the left. (D) H3unmod. and H3K9me3 chromatin templates were analyzed by western blotting using the indicated antibodies. (TIF) [file pone.0015894.s001.tif]

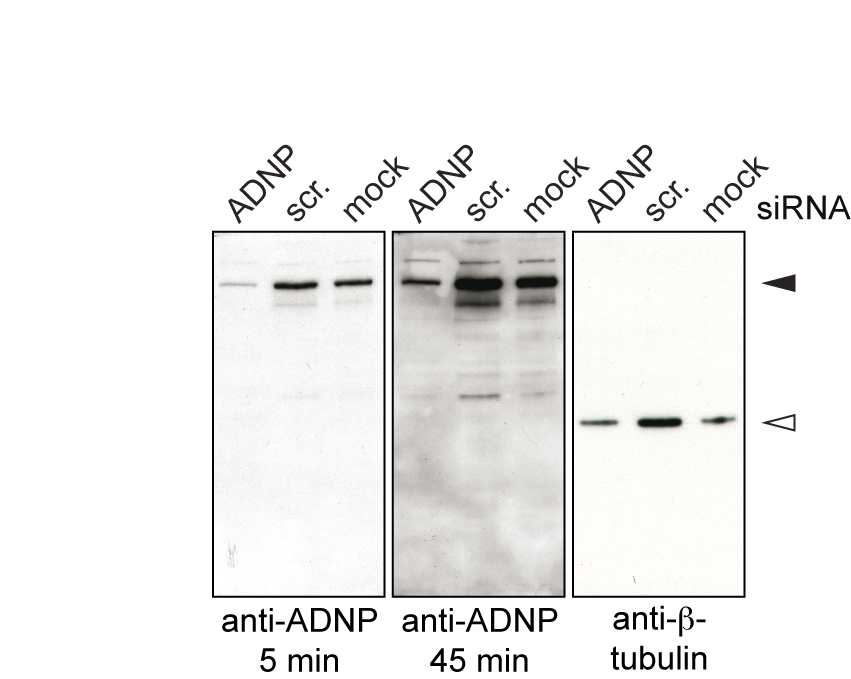

Supplement: Figure S2 — Specificity of the anti-ADNP monoclonal antibody. Extracts prepared from NIH3T3 cells treated with ADNP siRNA, corresponding scrambled siRNA (scr.) or untransfected (mock) were analyzed by western blotting using anti-ADNP monoclonal antibody or anti-β-tubulin antibodies. Different exposures of the ECL secondary antibody detection reaction are shown. Black arrowhead marks running position of ADNP; open arrowhead marks running position of β-tubulin. (TIF) [file pone.0015894.s002.tif]

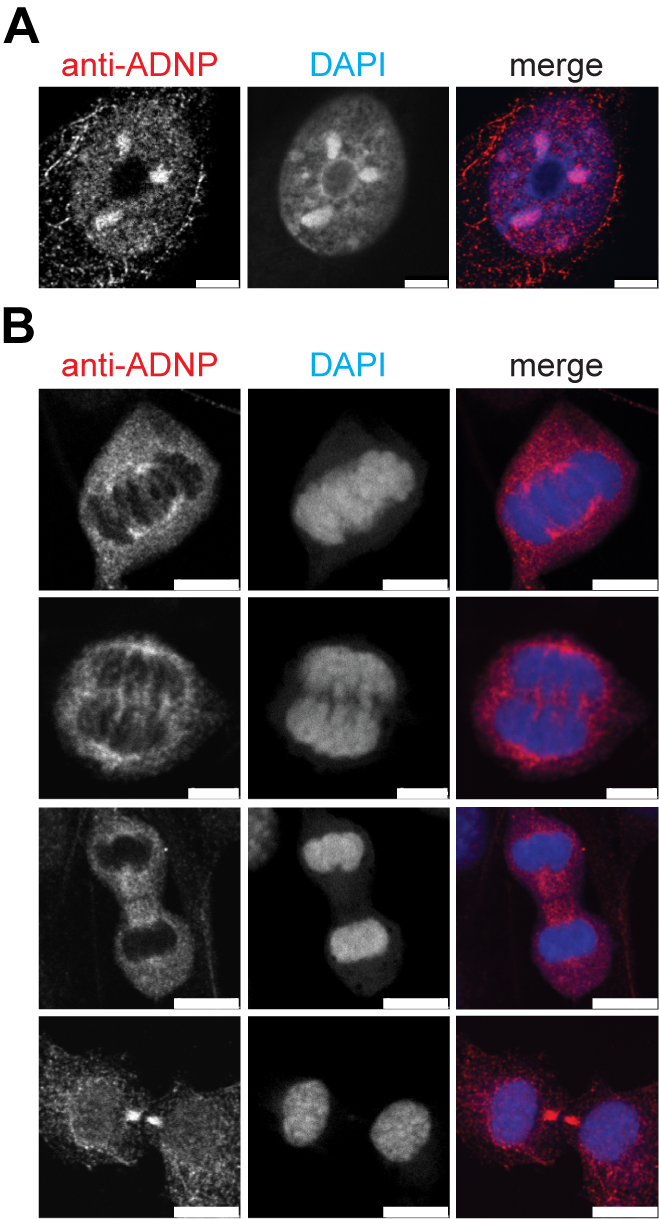

Supplement: Figure S3 — Localization of ADNP during the cell cycle. Immunofluorescence analysis of ADNP in NIH3T3 cells at interphase (A) and during M-phase (B). DNA was visualized using DAPI. Bars, 5 µm (A and B, lane 2) or 10 µm (B, rows 1, 3–4). (TIF) [file pone.0015894.s003.tif]

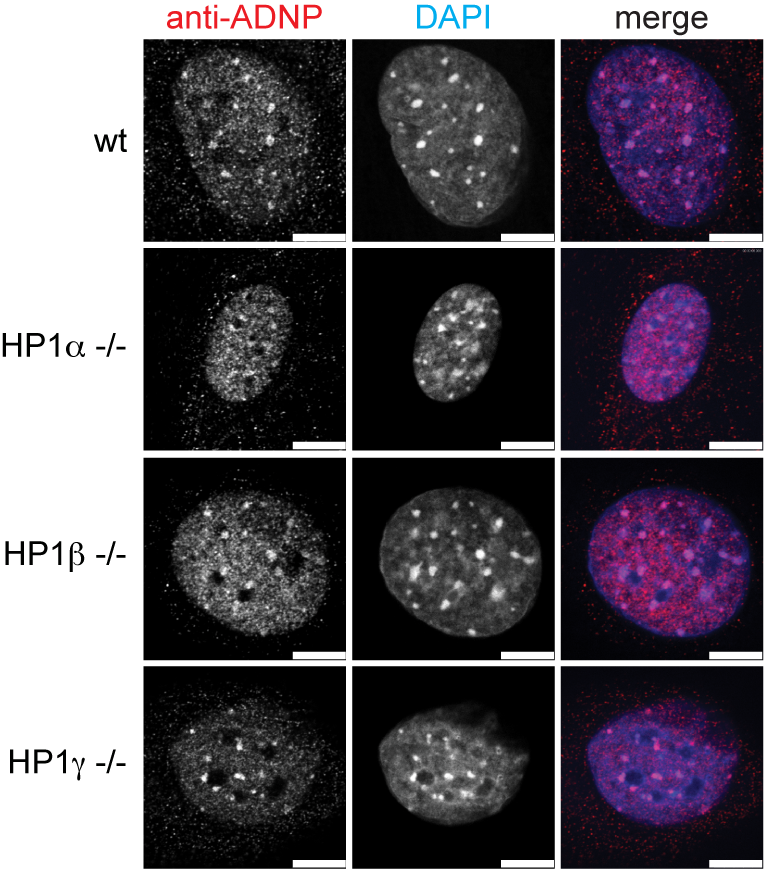

Supplement: Figure S4 — Absence of single HP1 isoform proteins does not affect ADNP localization to pericentromeric heterochromatin. Immunofluorescence analysis of ADNP in wild type (wt) or mutant MEF cells of the indicated genomic background after knock out of the indicated HP1 genes. DNA was visualized using DAPI. Bars, 7.5 µm. (TIF) [file pone.0015894.s004.tif]

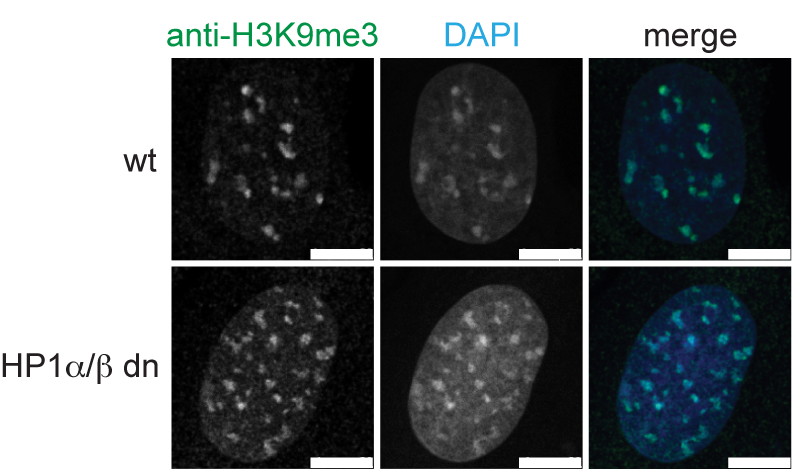

Supplement: Figure S5 — Absence of HP1α and HP1β does not affect localization of H3K9me3. Immunofluorescence analysis of H3K9me3 in MEF cells derived from HP1αHP1β double knockout mice (HP1αβdn). DNA was visualized using DAPI. Bars, 7.5 µm. (TIF) [file pone.0015894.s005.tif]

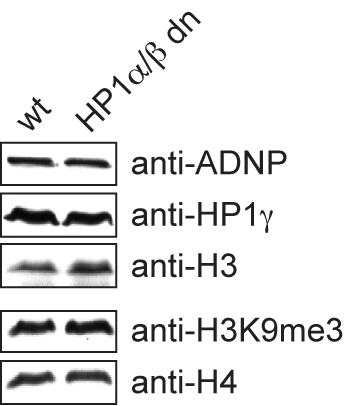

Supplement: Figure S6 — Absence of HP1α and HP1β does not affect ADNP, HP1γ or H3K9me3 levels. Western blot analysis of total cell extracts from wild type (wt) and HP1αHP1β double knockout (HP1αβdn) MEF cells using the indicated antibodies. (TIF) [file pone.0015894.s006.tif]

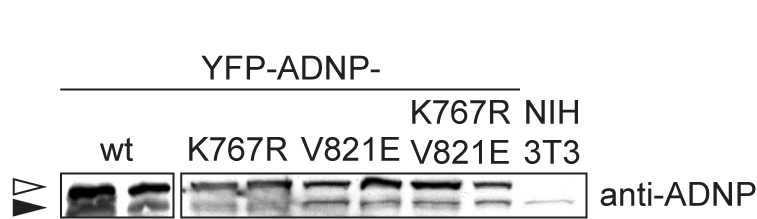

Supplement: Figure S7 — Expression levels of YFP-ADNP stable transfected cell lines. Western blot analysis of untransfected NIH3T3 cells or NIH3T3 cells stably expressing wild type YFP-ADNP (wt) or the indicated single or double mutant fusion proteins using the anti-ADNP antibody. The black arrowhead indicates the running position of endogenous ADNP; the open arrowhead indicates the running position of the YFP-ADNP fusion proteins. (TIF) [file pone.0015894.s007.tif]

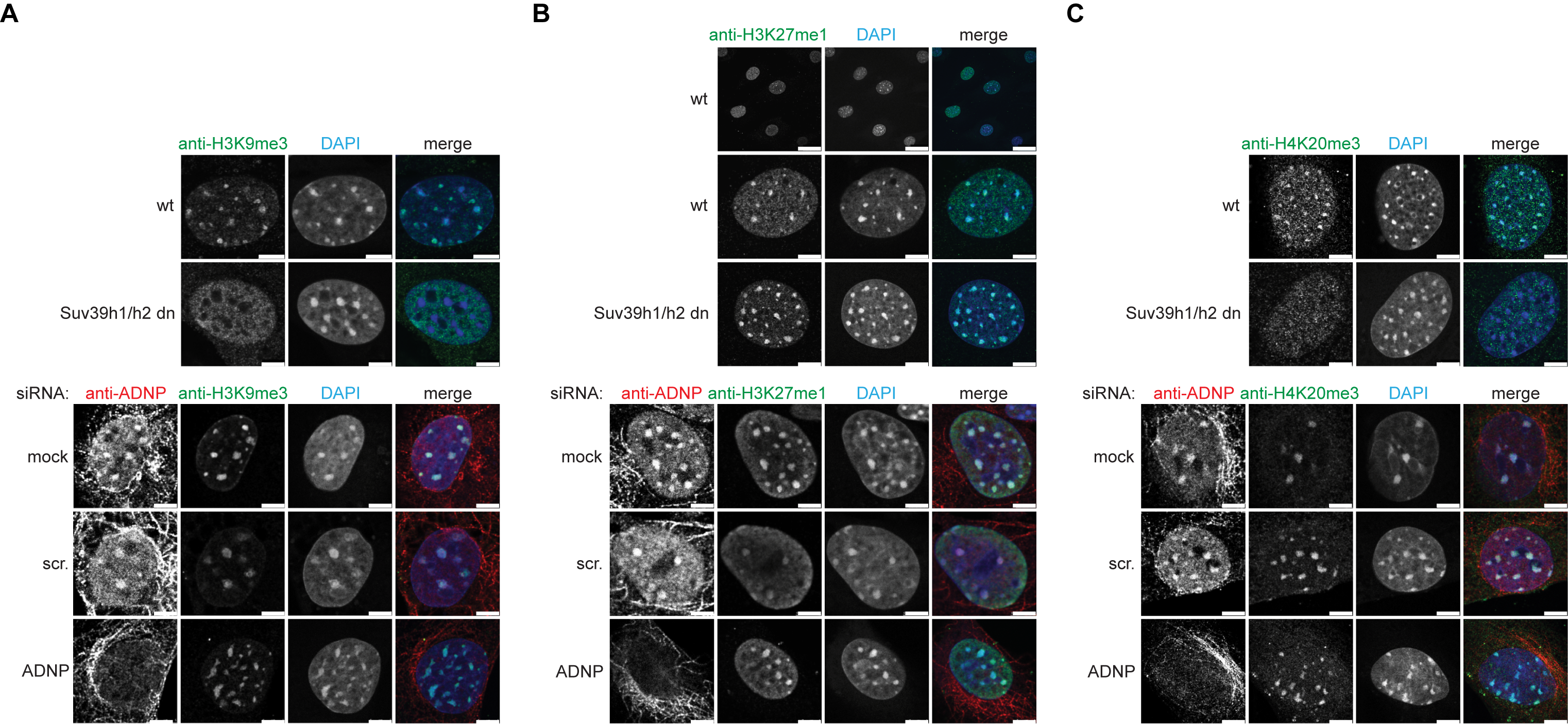

Supplement: Figure S8 — Nuclear distribution of H3K9me3, H3K27me1 and H4K20me3 is not affected by ADNP knockdown. Immunofluorescence analysis of H3K9me3 (A), H3K27me1 (B) and H4K20me3 (C) in wild type (wt) and Suv39h1,Suv39h2 double knockout (Suv39h1/h2 dn) MEF cells (top). Immunofluorescence analysis of H3K9me3 (A), H3K27me1 (B) and H4K20me3 (C) in untreated (mock) and NIH3T3 cells transfected with scrambled or ADNP targeting siRNAs (bottom). DNA was visualized using DAPI. Bars, 25 µm (B, upper row) or 5 µm. (TIF) [file pone.0015894.s008.tif]

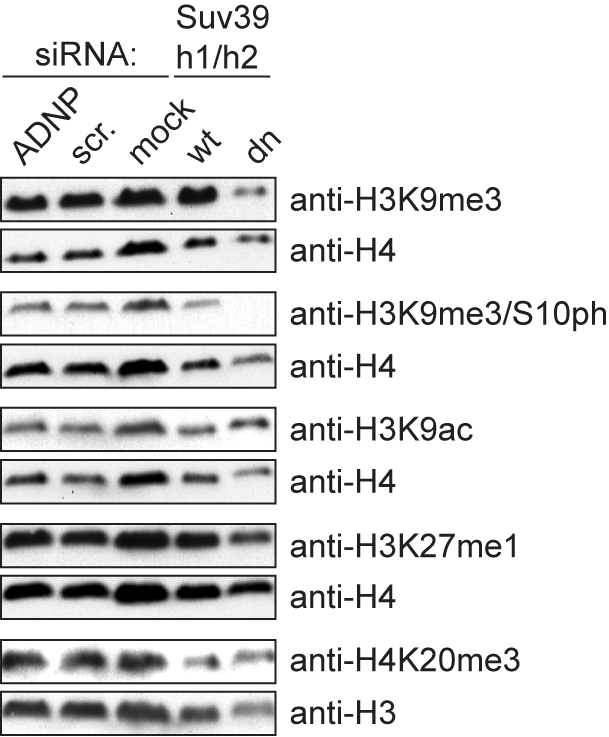

Supplement: Figure S9 — Levels of histone modifications in ADNP knockdown NIH3T3 cells are not changed. Western blot analysis of untreated (mock) and NIH3T3 cells transfected with scrambled (scr.) or ADNP targeting siRNAs as well as wild type (wt) and Suv39h1, Suv39h2 double knockout (Suv39h1/h2 dn) MEF cells using the indicated antibodies. (TIF) [file pone.0015894.s009.tif]

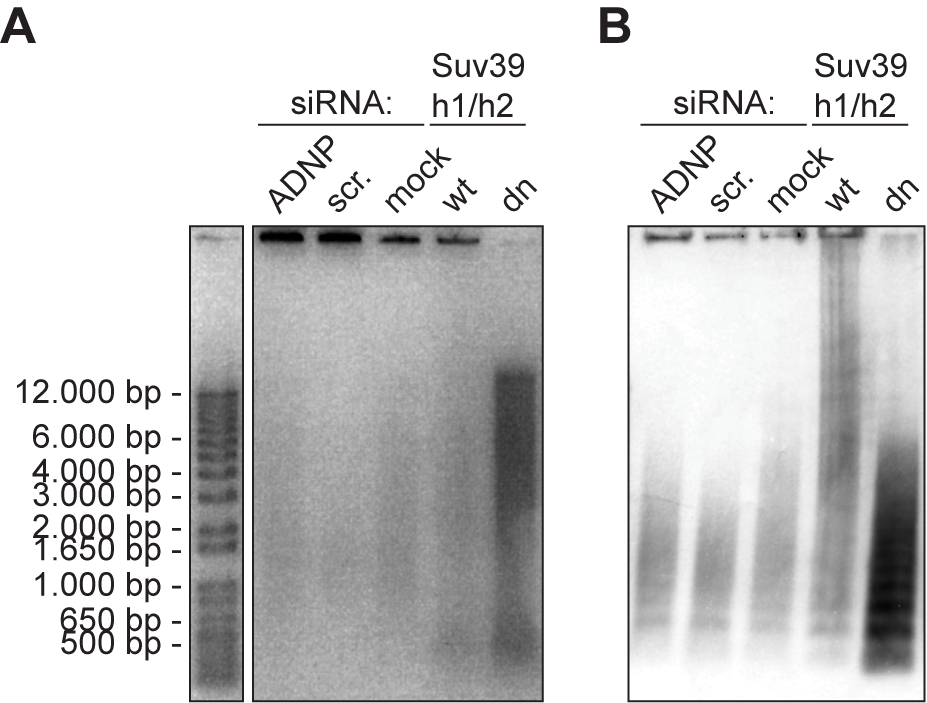

Supplement: Figure S10 — Knockdown of ADNP does not affect DNA methylation levels. Genomic DNA of untreated (mock) and NIH3T3 cells transfected with scrambled (scr.) or ADNP targeting siRNAs as well as wild type (wt) and Suv39h1, Suv39h2 double knockout (Suv39h1/h2 dn) MEF cells was digested with the restriction enzyme TaiI and separated on a 1% agarose gel. Ethidium bromide staining of the gel (A) and Southern blot (B) using a major satellite repeat probe are shown. (TIF) [file pone.0015894.s010.tif]

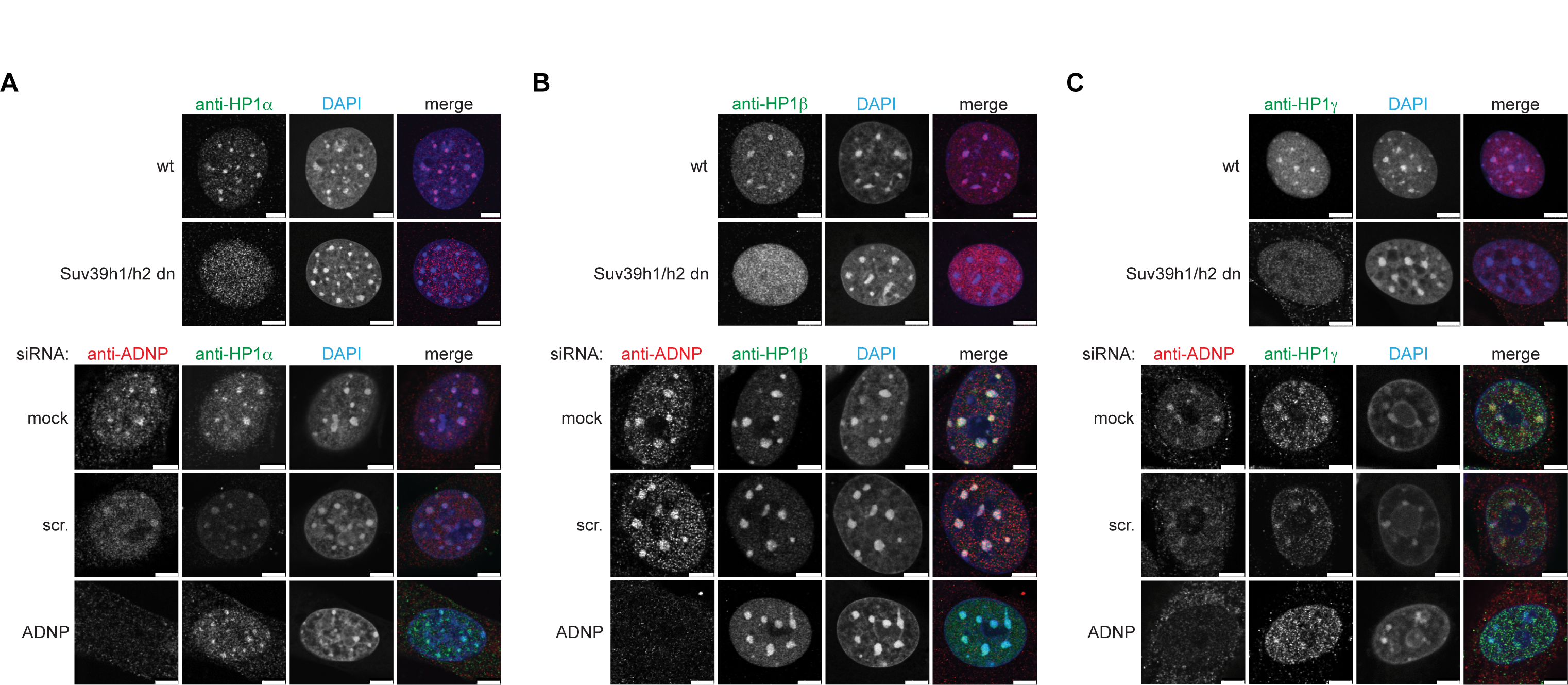

Supplement: Figure S11 — Nuclear distribution of HP1α, HP1β and HP1γ is not affected by ADNP knockdown. Immunofluorescence analysis of HP1α (A), HP1β (B) and HP1γ (C) in wild type (wt) and Suv39h1, Suv39h2 double knockout (Suv39h1/h2 dn) MEF cells (top). Immunofluorescence analysis of HP1α (A), HP1β (B) and HP1γ (C) in untreated (mock) and NIH3T3 cells transfected with scrambled (scr.) or ADNP targeting siRNAs (bottom). DNA was visualized using DAPI. Bars, 5 µm. (TIF) [file pone.0015894.s011.tif]

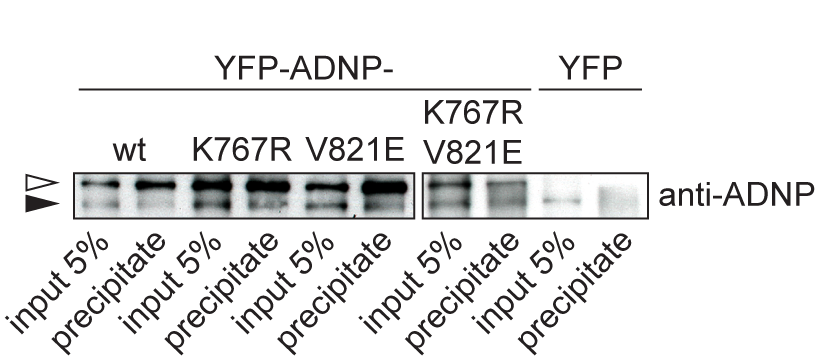

Supplement: Figure S12 — Mutation of the ADNP homeodomain does not affect protein multimerization. The indicated YFP-ADNP fusion proteins or YFP were immunoprecipitated from nuclear extracts of the corresponding stable transfected NIH3T3 cell lines using anti-GFP-antibodies. Western blot analysis of the immunoprecipitated (precipitated) material using the anti-ADNP antibody is shown. The black arrowhead indicates the running position of endogenous ADNP; the open arrowhead indicates the running position of the YFP-ADNP fusion proteins. (TIF) [file pone.0015894.s012.tif]

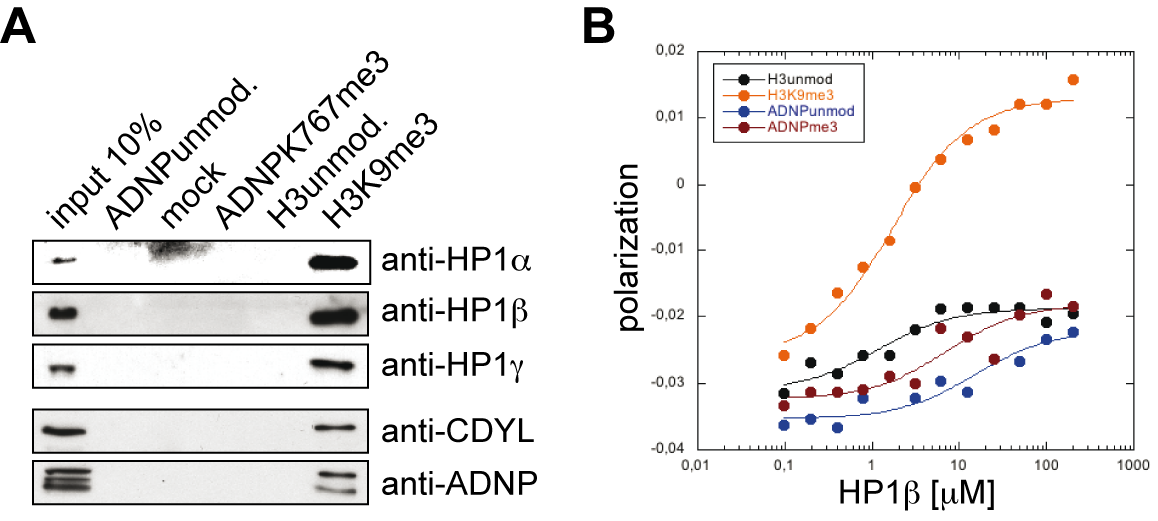

Supplement: Figure S13 — HP1 does not bind to ADNP K767me3. (A) The indicated ADNP and histone H3 peptides were used in pulldown experiments of HeLa S3 cell nuclear extract. Beads without coupled peptides were used as control (mock). Specifically recovered proteins were analyzed by western blotting using the indicated antibodies. (B) Fluorescence polarization binding assay of recombinant HP1β using the indicated H3 (unmodified, K9me3) and ADNP (unmodified, K767me3) peptides. The averaged fluorescence polarization signal from three independent titration reactions is shown. (TIF) [file pone.0015894.s013.tif]
